# Clinical Evaluation of Patients With Premature Canities and Its Association With Metabolic Syndrome

**DOI:** 10.7759/cureus.117274

**Published:** 2026-10-01

**Authors:** Hritik V Shah, Aneet Mahendra, Sanjeev Gupta, Aayush Goel, Aditi Dabhra

**Affiliations:** 1 Dermatology, Maharishi Markandeshwar Institute of Medical Sciences and Research (MMIMSR), Mullana, IND

**Keywords:** canities, graying of hair, metabolic syndrome (mets), premature graying of hair, trichology

## Abstract

Introduction: Premature canities (early hair graying) are no longer just a cosmetic concern; they are visible markers of systemic oxidative stress. The underlying depletion of follicular melanocytes mirrors the physiological aging associated with metabolic syndrome. Consequently, early graying can serve as a crucial preclinical warning sign for underlying cardiovascular risks, including central obesity, hypertension, and dyslipidemia.

Aims and objectives: This study aimed to investigate the clinico-epidemiological profile of patients with premature canities and to assess the prevalence of metabolic syndrome in these individuals compared with healthy controls.

Materials and methods: An observational case-control study, conducted at a tertiary care center, included 240 age- and sex-matched participants (120 cases and 120 controls). After collecting comprehensive demographic data, subjects were divided into mild, moderate, or severe categories based on the extent of hair graying at presentation. Metabolic syndrome parameters were subsequently evaluated using the NCEP ATP III (National Cholesterol Education Program Adult Treatment Panel III) criteria, which included waist circumference, blood pressure, fasting triglycerides, high-density lipoprotein, and fasting blood sugar.

Results: The study identified a highly significant male predominance among the participants (n=85, 70.8%). The mean age of onset for premature graying was notably early, at 16.58 ± 4.20 years. A positive family history was reported by 38.3% (n=46) of the participants. Anthropometric and biochemical evaluations revealed that cases exhibited a significantly higher mean waist circumference compared with controls (84.44 cm vs. 81.75 cm; p=0.020) and a higher mean systolic blood pressure (123.63 mmHg vs. 119.35 mmHg; p=0.001). Furthermore, lipid profiles in cases demonstrated significantly lower protective HDL cholesterol (50.10 mg/dL vs. 53.34 mg/dL; p=0.045) and elevated triglycerides (115.98 mg/dL vs. 103.40 mg/dL; p=0.023) compared with controls.

## Introduction

In today's visually driven world, we have given the utmost importance to hair. Far more than a mere biological feature, it is our crowning glory and a marker of identity. A person's personality, vitality, and confidence are often instantly perceived through the lush density, vibrant color, and perfect styling of their hair. While natural graying is universal, premature canities, clinically defined as graying before the age of 25 in Asians [1], a threshold and clinical picture that has been extensively characterized in comprehensive dermatological reviews of the condition [2], often trigger immense psychological distress for young adults, mourning the early loss of their youthful aesthetic.

Modern dermatology increasingly views this premature loss of pigment not merely as an isolated cosmetic frustration but as a visible warning signal from the body. It is heavily driven by systemic oxidative stress, which depletes follicular antioxidant defenses and damages melanocyte DNA, ultimately halting melanin synthesis within the hair bulb [3,4]. Crucially, this exact same oxidative stress is a primary trigger for metabolic syndrome, which is a dangerous cluster of health conditions that includes central obesity, high blood pressure, elevated blood sugar, and abnormal cholesterol levels [5]. This relationship is increasingly understood to be bidirectional, with systemic oxidative stress now recognized as both a driver and a consequence of the metabolic syndrome itself [6]. Emerging clinical evidence reveals a striking link, suggesting that individuals with underlying metabolic imbalances often go gray earlier than their healthy peers [7]. Therefore, the primary goal of this study is to explore the clinico-epidemiological profile of young patients experiencing premature graying.

## Materials and methods

An observational case-control study was conducted for a period of 12 months from March 15, 2025, to March 15, 2026, at the Dermatology OPD of Maharishi Markandeshwar Institute of Medical Sciences and Research, Mullana, Ambala, enrolling 240 subjects to evaluate the link between premature hair graying and metabolic syndrome. The participants were divided into 120 cases comprising individuals aged 6 to 25 with premature canities and 120 age- and sex-matched healthy controls. The sample size (n) was determined using the formula $n = \frac{Z^{2} \times P \times Q}{L^{2}}$, where Z is the standard normal deviate (1.96 for 95% confidence), P = 50% (assuming maximum variability in the absence of a definitive prior prevalence estimate), Q = 50%, and L = 9% (absolute allowable error), yielding a minimum sample size of 119, rounded to 120.

Subjects with a history of vitiligo or prior use of topical depigmenting agents (ammonia, hydrogen peroxide bleach) and industrial workers those with exposure to ammonia and hydrogen peroxide were strictly excluded. Patients were categorized into mild (5-20%), moderate (20-50%), and severe (>50%) groups based on the extent of gray hair at presentation.

All participants were assessed for metabolic syndrome using the NCEP ATP III (National Cholesterol Education Program Adult Treatment Panel III) criteria [5], in line with the diagnostic thresholds set out in contemporary American Heart Association/National Heart, Lung, and Blood Institute scientific statements [8], which evaluate five parameters: (1) waist circumference: >90 cm in men and >80 cm in women; (2) blood pressure: systolic >130 mmHg or diastolic >85 mmHg; (3) fasting triglycerides: >150 mg/dL; (4) high-density lipoprotein (HDL): <40 mg/dL in men and <50 mg/dL in women; and (5) fasting blood sugar (FBS): >100 mg/dL.

Along with this, the BMI (body mass index) of all participants was also calculated according to the formula weight (kg)/height (m²). Nutritional parameters were studied, which included levels of vitamin B12 and vitamin D3. Peripheral blood film samples were also observed among the cases and control group, including hemoglobin levels, to compare the blood picture between both groups.

Statistical comparisons were performed between the cases and controls with respect to nutritional and metabolic parameters. The significance of differences observed was determined by calculating p-values using the Student’s t-test for continuous variables and the chi-square test for categorical variables. All statistical analyses were carried out using Microsoft Excel (Microsoft Corporation, Redmond, Washington) software.

## Results

The study compared individuals with premature hair graying (cases) against age- and sex-matched healthy controls. Both groups had a strong male majority, consisting of 85 males (70.8%) and 35 females (29.2%), making a 2.42:1 male-to-female ratio. The average age was 19.44 ± 3.97 years for cases and 20.10 ± 3.30 years for controls. Table 1 depicts the distribution of cases and controls on the basis of their demographic profile.

**Table 1:** Distribution of subjects based on their demography Age group and sex are presented as frequency counts and percentages, n (%). Mean age is presented as mean ± standard deviation (SD). Pearson’s chi-square (χ²) test was used to analyze categorical variables (age group and sex). For the age group (3×2 contingency table), χ² = 1.361, df = 2, p = 0.137. For sex (2×2 contingency table), χ² = 0.000, df = 1, p = 1.000. The Student's independent-samples t-test was used to compare the continuous variable (mean age) between cases and controls (t = -1.401, df = 238, p = 0.163). Statistical significance: A p-value < 0.05 was set as the threshold for statistical significance. No statistically significant demographic differences were observed between cases and controls (p > 0.05), indicating well-matched study groups.

Demographic Characteristic	Cases (n = 120)	Control (n = 120)	t-value	Chi-square (χ²)	p-value
Age Group (years)					
6–12	6 (5.0%)	3 (2.5%)	-	1.361	0.137
13–19	50 (41.7%)	47 (39.1%)	-		
20–25	64 (53.3%)	70 (58.3%)	-		
Mean Age (± SD)	19.44 ± 3.97	20.10 ± 3.30	-1.401		0.163
Sex					
Male	85 (70.8%)	85 (70.8%)	-	-	1.000
Female	35 (29.2%)	35 (29.2%)	-	-	

In the current study, participants first noticed gray hairs at an average age of 16.58 ± 4.20 years. The majority (n=67, 55.8%) first spotted their gray strands during late adolescence (13-19 years). When evaluated, 58 (48.3%) cases presented with moderate graying (20-50 gray hairs), while 37 (30.8%) cases had severe graying (over 50 gray hairs).

A family history of early graying was a major risk factor, present in 46 (38.3%) cases compared to just 29 (24.1%) controls (p=0.026), with a notably higher familial history of systemic issues (p=0.0035), specifically thyroid disorders (23 cases vs. 11 controls) and diabetes mellitus (17 cases vs. 9 controls).

Smoking was nearly twice as common in the case group (27 (22.5%) vs. 14 (11.6%), p=0.038), and alcohol consumption was also significantly higher (14 (11.8%) vs. 4 (3.3%), p=0.022). Intensive cosmetic stressors (like keratinization, waxing, or chemical straightening) were more frequent in cases (27 (22.5%) vs. 15 (12.5%), p=0.041), with keratinization being the most common procedure.

Table 2 outlines the nutritional and hematological status of cases compared to controls (n=120 in each group), demonstrating highly significant deficiencies and red blood cell alterations among the cases. The cases displayed markedly lower levels of vitamin B12 (145.2 ± 42.5 pg/mL vs. 258.4 ± 55.3 pg/mL, P < 0.001) and vitamin D3 (16.8 ± 4.2 ng/mL vs. 27.5 ± 5.8 ng/mL, P < 0.001) than the control group. Hemoglobin levels were also significantly reduced in the cases at 11.4 ± 1.8 g/dL compared to 13.6 ± 1.2 g/dL in controls (P < 0.05). Furthermore, peripheral blood film (PBF) examinations revealed a substantial shift: a microcytic hypochromic pattern was prominent in 45.0% (n=54) of cases compared to just 11.6% (n=14) of controls, whereas the healthy normocytic normochromic status was present in 88.4% (n=106) of controls but dropped to 55.0% (n=66) among cases (P < 0.001).

**Table 2:** Nutritional and hematological parameters *P < 0.05 = statistically significant. Values are presented as mean ± SD or n (%). t-value = independent-samples t-test (for vitamin B12, vitamin D3, and hemoglobin). Chi-square value = chi-square test with Yates' correction (for PBF).

Parameter	Cases (n=120)	Controls (n=120)	t-value	Chi-square value	P-value
Vitamin B12 (pg/mL)	145.2 ± 42.5	258.4 ± 55.3	17.780	-	< 0.001*
Vitamin D3 (ng/mL)	16.8 ± 4.2	27.5 ± 5.8	16.368	-	< 0.001*
Hemoglobin (g/dL)	11.4 ± 1.8	13.6 ± 1.2	11.140	-	< 0.05*
Peripheral blood film (microcytic hypochromic)	54 (45%)	14 (11.6%)	-	31.211	< 0.001*
Peripheral blood film (normocytic normochromic)	66 (55%)	106 (88.4%)	-	31.211	< 0.001*

Table 3 compares various metabolic parameters between the cases and controls, revealing an elevated metabolic and cardiovascular risk profile for the cases. Cases presented with a significantly higher waist circumference (84.44 ± 7.77 cm vs. 81.75 ± 9.87 cm, P = 0.020) and elevated systolic BP (123.63 ± 9.96 mmHg vs. 119.35 ± 9.10 mmHg, P = 0.001), though diastolic BP showed no statistically significant difference between the two cohorts (79.74 ± 8.13 mmHg vs. 79.98 ± 6.80 mmHg, P = 0.810). Lipid profile was less favorable in the case group, marked by significantly lower cardioprotective HDL cholesterol (50.10 ± 12.32 mg/dL vs. 53.34 ± 12.61 mg/dL, P = 0.045) and significantly elevated triglycerides (115.98 ± 43.43 mg/dL vs. 103.40 ± 42.02 mg/dL, P = 0.023). Lastly, while fasting blood sugar variations remained statistically non-significant (94.48 ± 14.29 mg/dL in cases vs. 97.37 ± 19.56 mg/dL in controls, P = 0.193), the overall BMI was significantly higher in the cases at 24.8 ± 3.1 kg/m² compared to 21.4 ± 2.5 kg/m² for the controls (P < 0.05).

**Table 3:** Metabolic parameters comparison *P < 0.05 = statistically significant. Values are presented as mean ± SD. t-value = independent-samples t-test (used for all parameters in this table). BP = Blood Pressure; HDL = High-Density Lipoprotein; BMI = Body Mass Index.

Metabolic parameter	Cases (n=120)	Controls (n=120)	t-value	P-value
Waist Circumference (cm)	84.44 ± 7.77	81.75 ± 9.87	2.346	0.020*
Systolic BP (mmHg)	123.63 ± 9.96	119.35 ± 9.10	3.475	0.001*
Diastolic BP (mmHg)	79.74 ± 8.13	79.98 ± 6.80	0.248	0.810
HDL Cholesterol (mg/dL)	50.10 ± 12.32	53.34 ± 12.61	2.013	0.045*
Triglycerides (mg/dL)	115.98 ± 43.43	103.40 ± 42.02	2.280	0.023*
Fasting Blood Sugar (mg/dL)	94.48 ± 14.29	97.37 ± 19.56	1.307	0.193
BMI (kg/m²)	24.8 ± 3.1	21.4 ± 2.5	9.352	< 0.001*

In this study, 37 (30.8%) of the cases met three or more diagnostic criteria for metabolic syndrome (NCEP ATP III), compared to 26 (21.6%) of the healthy controls, a statistically significant difference (p=0.037).

## Discussion

Our hair is our ultimate accessory, a personal canvas we use to express who we are to the world. It dictates our daily confidence. As a society, we are captivated by the endless hair trends; those with straight hair spend hours curling it, while those with natural curls endure harsh chemical straightening. We highlight our black hair to catch the light, and the moment a white hair appears, we rush to dye it black. We treat our hair as an aesthetic playground, but beneath the dyes and all the styling procedures, our hair might just be trying to tell us a story about our internal health. Historically dismissed as a genetic trait [9], finding gray hair in our youth is now recognized as a possible marker of systemic oxidative stress associated with follicular melanocyte loss.

Premature canities is far from a simple cosmetic defect; it is a multi-systemic marker where established endocrine susceptibilities and nutritional deficiencies converge with previously unexplored metabolic risks. Similar to other studies, our study confirms a traditional foundation of genetic and endocrine vulnerability, highlighted by high familial rates of thyroid abnormalities and diabetes; this pattern has also been seen in other studies [10,11]. The study also validates the significant depletion of essential micronutrients like vitamins B12 and D3 alongside widespread iron-deficiency anemia [12]; its most notable finding lies in the largely unexamined metabolic frontier. Our study demonstrates that early pigment loss, which was most pronounced in young men around the age of 16, serves as a visible marker, especially for early metabolic syndrome complications, as seen by various studies that show unfavorable upward trends in central obesity (waist circumference), systolic blood pressure, and serum triglycerides, paired with suppressed protective HDL cholesterol in cases compared to healthy controls [7]. This metabolic shift is further substantiated by a significantly higher average body mass index in our cases; while visceral central adiposity remains a more specific diagnostic criterion for metabolic syndrome, this elevated BMI stands as an important parameter that confirms a clear link between overall weight status and accelerated hair graying [13]. As fasting blood sugar levels remained statistically similar between both groups, this normoglycemic state highlights a vital developmental timeline, positioning premature graying as an early warning sign that manifests during the silent phases of metabolic decline right before the formal onset of type 2 diabetes. Accelerated by modern oxidative stressors like smoking and chemical treatments that destroy follicular melanocytes, premature gray hair ultimately acts as a unique, macroscopic clinical window, offering clinicians a timely opportunity to intervene proactively and monitor a young patient's long-term metabolic trajectory, as seen in various studies [14,15].

In our study, we compared 120 people with early graying against 120 controls; we found that 54 of the premature graying group had nutritional deficiencies compared to just 14 in the healthy group, and 37 cases had metabolic syndrome compared to 26 controls (none of the controls had any clinical symptom pointing towards metabolic syndrome). With 20 individuals dealing with both metabolic and nutritional abnormalities, it is clear that early graying is not triggered by just one factor; it is actually a complex interconnection of nutritional, metabolic, and genetic factors. While many studies have pointed to a link with poor nutrition, the link to metabolic health is unexplored. This shows that premature graying could actually serve as a vital marker for underlying systemic health issues, making large-scale future studies incredibly important to find out this connection.

The current study had a few constraints and limitations, such as the small sample size; a subjective scale was used for grading the severity of premature canities. Trichoscopic evaluation of the severity of graying or a modified phototrichogram could not be used in this study, and as the questionnaire was self-administered, there was a possibility of recall bias.

## Conclusions

Premature hair graying should fundamentally no longer be dismissed as merely an aesthetic inconvenience or genetic inevitability. It serves as a profound, visible biological indicator of internal aging and widespread systemic oxidative stress. This comprehensive study concretely links the early onset of canities to parameters of metabolic syndrome, specifically central adiposity, systolic hypertension, and dyslipidemia (elevated triglycerides and lowered HDL). By observing these markers in patients frequently still in their late teens, the hair follicle proves itself to be an invaluable, non-invasive clinical tool. By recognizing premature graying as a clinical indicator of metabolic abnormalities, dermatologists possess an early window of opportunity. During this preclinical phase, immediate preventive lifestyle modifications can be implemented, offering an opportunity to monitor long-term metabolic and cardiovascular parameters and prevent their full-scale onset.
